# Supplementary material for: Beyond Taxonomic Identification: Integration of Ecological Responses to a Soil Bacterial 16S rRNA Gene Database
Source: Front Microbiol. 2021 Jul 19;12:682886. doi: 10.3389/fmicb.2021.682886 (PMC8326369; doi:10.3389/fmicb.2021.682886)
Supplement: Supplementary Figure 1 — ID-TaxER database Infrastructure 16S sequences are queried over the web via the R Shiny interface. A BLAST search is then performed against a blast database containing representative 16S sequences from the 2007 Countryside survey. Model information and associated metadata for match hits are located in a PostgreSQL database of OTU taxonomy/model data (model objects are stored as binary and retrieved for the user) and results displayed via the shiny interface. [file Table_1.DOCX]

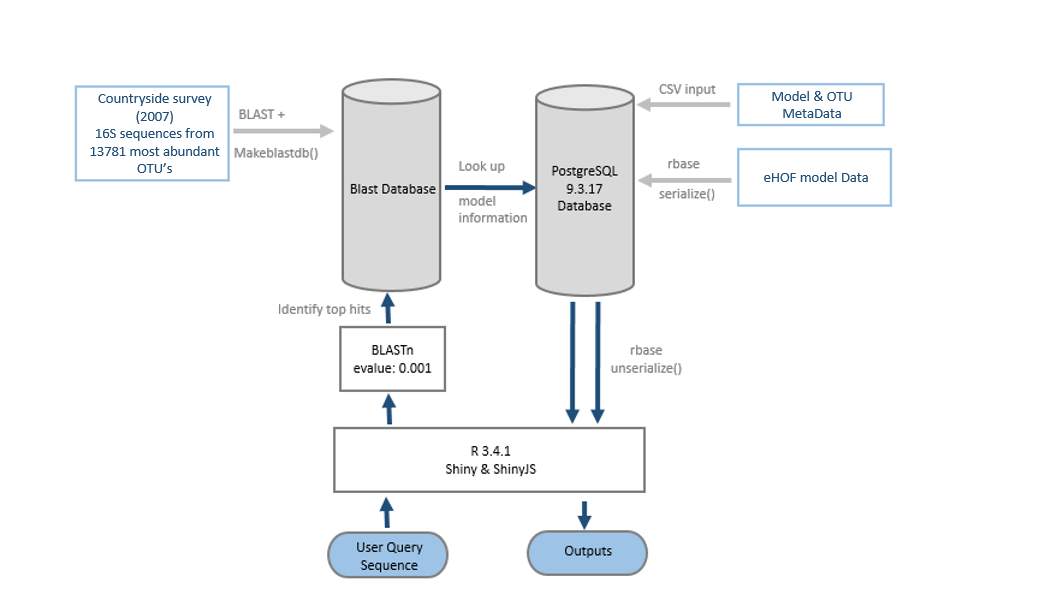


**Supp.fig.1 ID-TaxER database Infrastructure** 16S sequences are queried over the web via the R Shiny interface. A BLAST search is then performed against a blast database containing representative 16S sequences from the 2007 Countryside survey. Model information and associated metadata for match hits are located in a PostgreSQL database of OTU taxonomy/ model data, (model objects are stored as binary and retrieved for the user) and results displayed via the shiny interface.


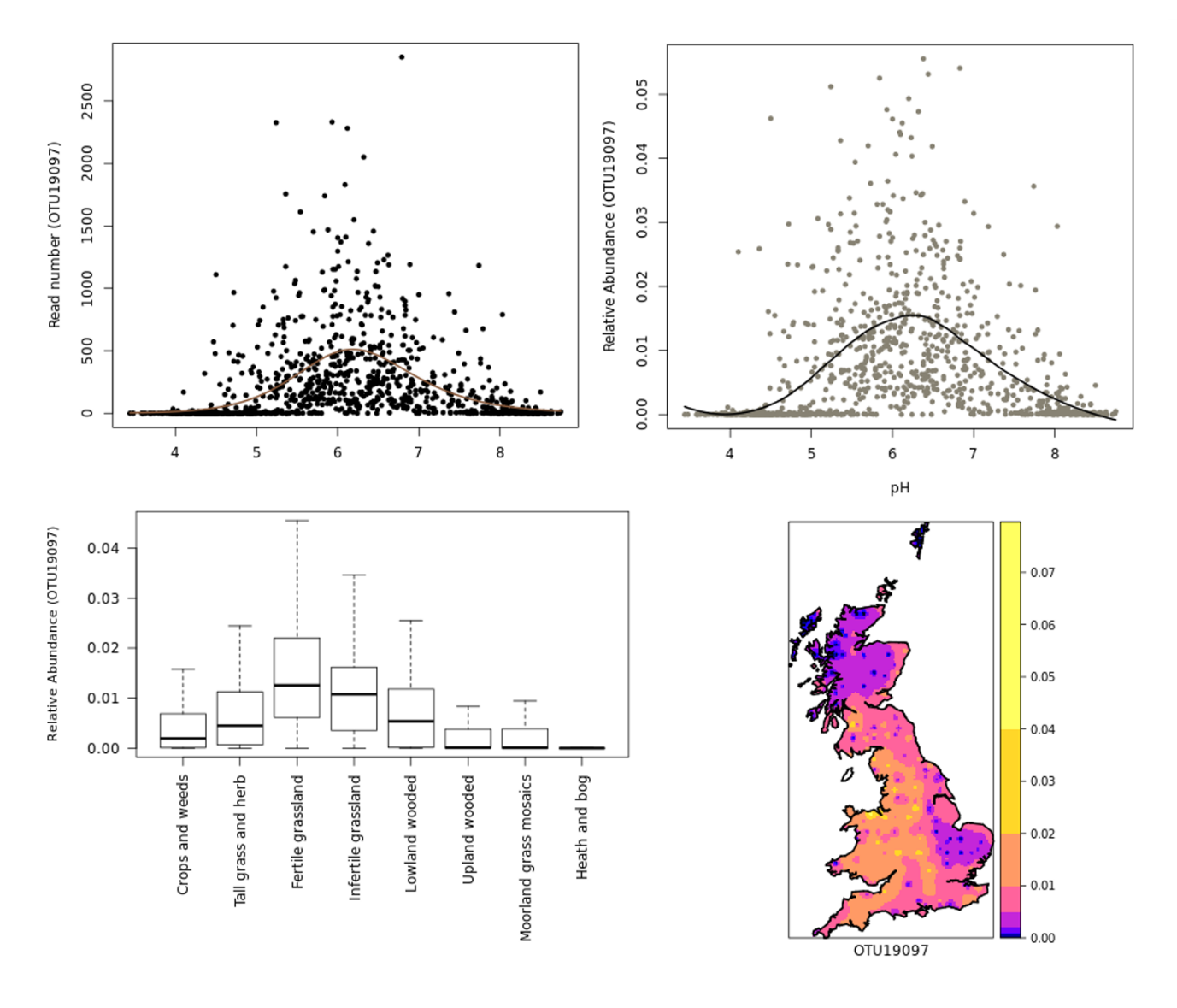


**Supp.fig.2 Example outputs from the ID-TaxER online portal.**  Using the DA101 /Ca. U. copiosus**^42^** 16S sequence (GenBank: Y07576.1) as a query, we found 98.3% identitiy to CS OTU19097 (taxonomy=k_Bacteria; p_Verrucomicrobia; c_Spartobacteria; o_Chthoniobacterales; f_Chthoniobacteraceae; g_DA101): a) HOF model output showing the number of reads of CS OTU19097 per sample plotted against soil pH; with the line representing the model fit ( Model V, unimodal response to pH with an optima at pH 6.18) b) the relative abundance of OTU19097 against sample pH, with the line representing a LOESS fit; c) boxplot showing the median and ranges of the relative abundance of OTU19097 per CS habitat class; d) inverse distance weighted interpolation map of the relative abundance of OTU19097 across Britain.
